# The uPAR System as a Potential Therapeutic Target in the Diseased Eye

**DOI:** 10.3390/cells8080925

**Published:** 2019-08-18

**Authors:** Maurizio Cammalleri, Massimo Dal Monte, Vincenzo Pavone, Mario De Rosa, Dario Rusciano, Paola Bagnoli

**Affiliations:** 1 Dipartimento di Biologia, Università di Pisa, 56127 Pisa, Italy; 2 Dipartimento di Scienze Chimiche, Università di Napoli Federico II, 80126 Napoli, Italy; 3 Dipartimento di Medicina Sperimentale, Università della Campania, 80138 Napoli, Italy; 4 Sooft Italia SpA, Contrada Molino 17, 63833 Montegiorgio (FM), Italy

**Keywords:** ocular diseases, animal models, angiogenesis, inflammation, vascular leakage, photoreceptor degeneration, retinal function, co-receptor signaling, uPAR system blockade

## Abstract

Dysregulation of vascular networks is characteristic of eye diseases associated with retinal cell degeneration and visual loss. Visual impairment is also the consequence of photoreceptor degeneration in inherited eye diseases with a major inflammatory component, but without angiogenic profile. Among the pathways with high impact on vascular/degenerative diseases of the eye, a central role is played by a system formed by the ligand urokinase-type plasminogen activator (uPA) and its receptor uPAR. The uPAR system, although extensively investigated in tumors, still remains a key issue in vascular diseases of the eye and even less studied in inherited retinal pathologies such as retinitis pigmantosa (RP). Its spectrum of action has been extended far beyond a classical pro-angiogenic function and has emerged as a central actor in inflammation. Preclinical studies in more prevalent eye diseases characterized by neovascular formation, as in retinopathy of prematurity, wet macular degeneration and rubeosis iridis or vasopermeability excess as in diabetic retinopathy, suggest a critical role of increased uPAR signaling indicating the potentiality of its modulation to counteract neovessel formation and microvascular dysfunction. The additional observation that the uPAR system plays a major role in RP by limiting the inflammatory cascade triggered by rod degeneration rises further questions about its role in the diseased eye.

## 1. Introduction

**Figure 1:**
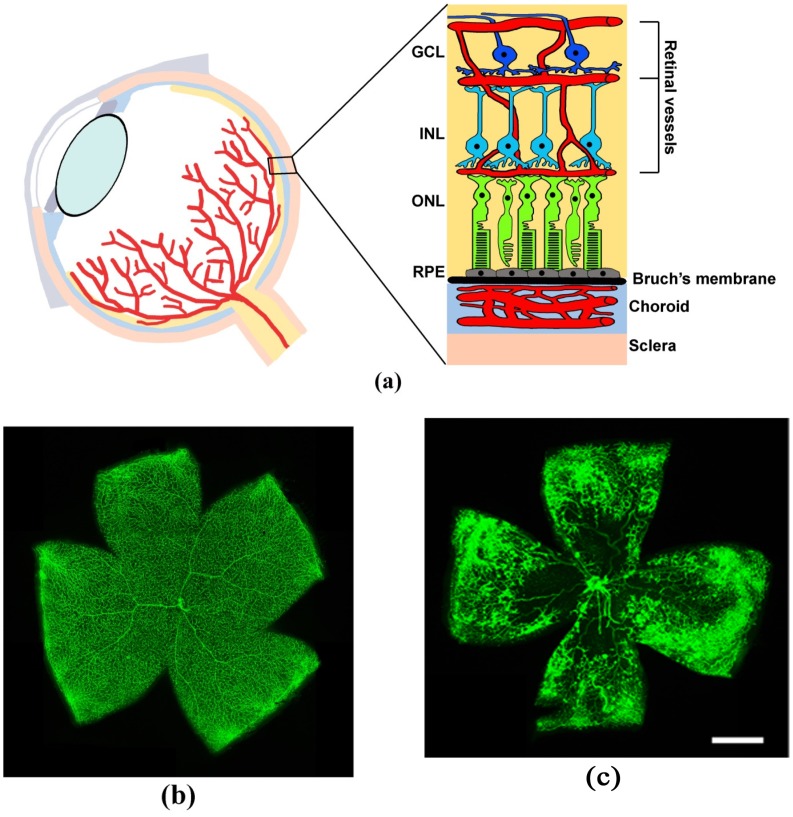
Schematic representation of a coronal section through the eye and retinal whole mounts. (**a**) Scheme of the eye: sectional view of retina layered structure and its vasculature. Only the three main classes of principal neurons—photoreceptors (green), bipolar cells (light blue), ganglion cells (blue) —are shown together with choroidal and retinal vasculature. Note that retinal vessels form three distinct plexuses, one in the inner part of the ganglion cell layer (GCL, superficial plexus) and the other two lining both sides of the inner nuclear layer (INL, intermediate and deep plexuses, respectively). RPE, retinal pigment epithelium; ONL, outer nuclear layer. (**b**,**c**) Representative retinal whole mounts showing the superficial plexus in control (**b**) and hypoxic conditions that refer to the oxygen induced retinopathy (OIR) model (**c**). In the OIR model, mice are exposed to hyperoxia from postnatal day (PD)7 to PD12, which leads to the arrest or retardation of the normal development of the retinal vasculature. When the animals are returned to normoxia, they experience a relative hypoxia especially in those retinal regions where normal vasculature is lacking. This situation results in unregulated, abnormal neovascularization occurring in the mid-peripheral retina. (**b**,**c**) are from unpublished material. To prepare the images, retinas were collected from either normoxic (**b**) or OIR (**c**) mice at PD17 and were immersion fixed in 4% paraformaldehyde in phosphate buffer. Retinas were then processed following standard immunohistochemical protocols using a rat monoclonal antibody directed to cluster of differentiation (CD) 31 (BD Pharmingen, San Diego, CA, USA), an endothelial cell marker, at 1:50 dilution and an Alexa Fluor 488 (Molecular Probes, Eugene, OR, USA) conjugated secondary antibody at 1:200 dilution. Scale bar: 1 mm.

The intricate functional coupling between retinal neurons, their supporting cells (astrocytes and Müller glial cells), and the vascular beds (endothelial cells and pericytes) work in close coordination in order to integrate vascular flow with retinal metabolic activity. As a result of correct relationships, a well-functioning blood-retinal barrier (BRB) is established to create an appropriate environment that contributes to correct visual function (for Ref., see [1]). Figure 1 shows a schematic representation of a coronal section through the eye depicting retinal circuitry and ocular vasculature together with retinal whole mounts showing the superficial plexus in normal and hypoxic conditions.

Altered functional and structural relationships between glial, neuronal and vascular cells may be recognized in several retinal pathologies in which capillary integrity is compromised thus leading to dysfunctional BRB and increased vascular leakage, eventually accompanied by the development of new blood vessels.

The understanding of the molecular mechanisms underlying vascular diseases of the eye has increased vastly during the last decades and a myriad of angiogenic agents, across multiple families, have been identified. An imbalance in favor of pro-angiogenic factors stimulates angiogenesis and vasopermeability excess, but inflammation has also been recognized as a major component of vascular dysfunction of the eye. In a first phase, inflammation acts as a defense mechanism to maintain tissue homeostasis. However, sustained inflammation can be detrimental to tissue integrity, thus becoming a feature in the pathogenesis of vascular diseases (for Ref., see [2]).

Altered vascular patterning lays the ground to the accumulation of pro-angiogenic and inflammatory factors producing a detrimental environment that may lead to retinal cell death and altered function. Nevertheless, there are retinal neurodegenerative diseases that strictly depend on neuroinflammatory events without the involvement of angiogenic processes. In this respect, retinal diseases in which photoreceptors degenerate are even characterized by an anti-angiogenic state and a decreased retinal vascularization due to the hyperoxic environment generated by the reduced oxygen consumption consequent to photoreceptor death.

Mechanisms underlying the complex relationships between angiogenic processes, inflammation and retinal cell degeneration include multiple interconnected signaling pathways [3]. Among these pathways, a role for the urokinase-type plasminogen activator receptor (uPAR) system as a key player at the intersection between angiogenesis, inflammation and neurodegeneration has recently emerged. The uPAR system has been evidenced to act in promoting angiogenesis and inflammation in models of vascular pathologies of the eye and its inhibition leads to ameliorative effects of the pathological state. In addition, inhibiting the uPAR system has been revealed as a promising strategy in delaying cone death in a model of inherited rod degeneration with a prominent inflammatory component. Overall, experimental findings point to the uPAR system as a promising target to counteract a broad range of clinically relevant eye disorders.

Here, we will review the role of the uPAR system in eye diseases including its implication on pathologic angiogenesis, vasopermeability excess and photoreceptor loss in order to underline the link between ischemia and inflammation.

## 2. Degenerative Diseases of the Retina

Vision is the primary sense for humans. Therefore, understanding mechanisms underlying retinal degeneration is a goal to both prevent and counteract blinding diseases. Among degenerative diseases of the retina, ischemic retinopathies are due to metabolic alterations of the retinal environment as in rubeosis iridis (RI), diabetic retinopathy (DR), retinopathy of prematurity (ROP) or age-related macular degeneration (AMD) [2,4] while retinal dystrophies are the consequence of genetic defects as in retinitis pigmentosa (RP) [5]. The first group of diseases is characterized by vasopermeability excess eventually leading to neovascularization, then resulting in retinal cell death (for Ref., see [6]). RP is characterized by photoreceptor degeneration associated with remodeling of blood vessels that undergo a process of progressive atrophy opposite to neovascularization typical of ischemia-associated degenerative pathologies [7]. Both groups of diseases share common chronic inflammatory processes that are mediated by Müller and microglial cells that, in response to retinal injuries, become activated, and the production and release of inflammatory cytokines increases [8,9].

Aberrant angiogenesis in ophthalmology spans from the anterior to the posterior segments of the eye. Pathological angiogenesis in the anterior segment of the eye includes iris neovascularization. RI, the clinical term for iris neovascularization, is generally a result of impaired pro-angiogenic stimulus from other ocular pathologies linked to the progression of proliferative retinopathy (PR). The imbalance in angiogenesis and inflammation factors in both the posterior and anterior chambers of the eye during PR progression stimulates iris vasculature to undergo neoangiogenesis [10]. Ultimately, RI can induce the obstruction of Schlem’s cannal resulting in elevated intraocular pressure that leads to neovascular glaucoma causing ganglion cell death and visual dysfunction [4]. In the posterior segment of the eye, angiogenesis-related ocular pathologies can be divided into retinal vascular diseases, in which there is leakage and/or neovascularization from retinal vessels, and subretinal neovascularization, in which new vessels grow into the normally avascular outer retina and subretinal space. The former comprises diseases such as proliferative DR (PDR) and ROP, while the latter includes the wet or neovascular form of AMD (nAMD) [11]. Of them, PDR is the advanced stage of DR, one of the main complications affecting patients with diabetes and a leading cause for vision loss in the Western world [12]. In the early phase of DR (or non-PDR), high blood sugar activates several complex interconnecting pathways including those producing pro-angiogenic and inflammatory factors that play a prominent role in increasing vascular permeability [13]. The initial microvascular phase may be already accompanied by retinal cell degeneration thus indicating that DR should be considered as a neurovascular degeneration, not merely a microvascular disease [14]. Alterations in retinal functionality are reflected by impaired neurotransmitter signaling, predominant apoptotic processes, and thinner nerve fiber layer that may occur at early preclinical stages in concomitance with still subtle changes in microvascular hemodynamics (for Ref., see [15]). Glial cell alterations also contribute to retinal damage although glial cell dysfunction may initially serve as an adaptive response to stress conditions [16]. Years after diabetes onset, the severity of non-PDR progresses and culminates in PDR. Breakdown of the BRB causes a leakage of plasma proteins thus forming exudates that may lead to diabetic macular edema (DME), which is a major cause of drastic loss of visual acuity. During DR progression, adaptive changes begin to fail leading to retinal vascular endothelial cell proliferation through the internal limiting membrane into the vitreous where proliferating vessels may cause vitreous hemorrhage and/or tractional retinal detachment. New vessel growth is also a feature of ROP, a proliferative retinal vascular pathology that affects pre-term infants with low birth weight and exposure to high oxygen supplementation. In most cases, ROP resolves without treatment, causing no damage, but advanced ROP can cause permanent vision problems or blindness. As premature births are increasing worldwide and the high standard of neonatal care leads to the ever-increasing survival rate of low and very low birth-weight infants, ROP has become a major cause of childhood blindness. The pathologic progress of ROP starts with arrest of immature retinal development of both vessels and neurons by premature birth (phase I), followed by tissue ischemia, thereby resulting in hypoxia-induced neovascularization (phase II). In addition to prematurity, hyperoxia (room oxygen level after birth compared with in utero, combined with supplemental oxygen) also contributes to the initial delay of vascular growth [17]. This is due to the suppression of oxygen-regulated angiogenic growth factors. As the retina grows with increased metabolic demands, ischemic retinas become hypoxic and drive increased angiogenic growth factors leading to the second proliferative phase, which may cause exudates, fibrous scar formation and tractional retinal detachment. Recent evidence suggest that inflammation may contribute to a gradual increase in the risk for ROP and that inflammatory factors play a central role in ROP progression (for Ref., see [2]). At the choroidal level, growth of new vessels and their invasion into the subretinal space characterizes nAMD, the most common cause of vision loss in the elderly. The initial stimulus for choroidal neovascularization (CNV) is still unraveled, yet a most favored model corresponds to synergistic local inflammation and chronic hypoxia that trigger the vascular ingrowth [18]. In particular, the imbalance in a multitude of pro-angiogenic and inflammatory factors, under the control of hypoxia-dependent and -independent transcription factors, leads to the invasion of the subretinal space by the newly formed vessels leading to exudation and acute vision loss.

In ischemic retinopathies, visual loss is likely to depend on vascular damage affecting, in turn, the viability of inner retinal neurons and glial cells, while in inherited photoreceptor degenerative diseases such as RP, retinal impairment depends on disrupted phototransduction. RP is a heterogeneous group of genetically inherited blinding disorders affecting photoreceptors in which rods are preferentially targeted first thus resulting in night vision loss. As the disease progresses, RP invariably evokes secondary cone photoreceptor loss that causes severe visual dysfunction. In addition to mutations in dozens of different genes, RP is indeed worsened by molecular mechanisms that are independent on gene mutations. For instance, a chronic inflammation, secondary to the primary genetic defect, leads to rod death. Gliotic events exacerbating inflammation appear to establish a positive feedback loop that subsequently leads to cone death strengthening retinal degeneration.

## 3. Animal Models Mimicking Eye Pathologies and Their Potential Value for Developing Novel Treatments

A large body of our knowledge on the molecular aspects of the pathogenesis of eye diseases stems from animal models although they do not fully reflect the complex human conditions, thus emphasizing the importance of epidemiological studies with an unbiased molecular dimension.

Among the animal models of ocular diseases, a model of puncture-induced iris neovascularization is used to mimic RI [19,20]. It is based on the induction of iris vascular response by a series of self-sealing uveal punctures on BALB/c mice, and takes advantage of the postpartum maturation of mouse ocular vasculature. Mouse pups are subjected every fourth day to uveal punctures from the day of eye opening, postnatal day (PD)12.5, until PD24.5. The RI model develops neovascularizarion independently on altered vascular endothelial growth factor (VEGF) signaling. In particular, puncture-induced iris angiogenesis is mainly driven by inflammatory and plasminogen activating systems [19]. A main consequence of RI is often neovascular glaucoma, a devastating ocular disease, commonly associated with the late stage of DR.

Early stages of DR are mimicked by several animal models that have been instrumental for studying their underlying mechanisms together with possible pharmacological interventions. However, no single animal model represents the complete range of vascular and neural complications of human DR in both early and late stages. The rodent model of streptozotocin-(STZ-) induced DR is used as a surrogate model of type 1 diabetes, in which pancreatic beta cells are destroyed by the toxic activity of STZ, thus leading to a quick onset of diabetes. After 2–4 weeks, this model very closely recapitulates the initial process of DR, including increased levels of pro-angiogenic and inflammatory factors, gliosis, BRB breakdown, and apoptosis of the inner retinal neurons, which can all contribute to the retinal dysfunction as detected by changes in the electroretinogram (ERG). However, this model does not cover the final proliferative phase [21] that is a typical finding in severe diabetic patients. Although neither macular edema nor proliferative disease ever develop in the STZ model, results on the efficacy of therapeutic agents have often been used as the sole preclinical data underlying clinical studies in DME patients [22]. Among the animal models of DR, the spontaneously diabetic Torii (SDT) rat exploited by Sasase [23] is an inbred rat strain isolated from an outbred colony of Sprague–Dawley rats that despite the chronic severe hyperglycemia, survives for a long time without insulin treatment and is characterized by late diabetes onset followed by DR eventually progressing to massive hemorrhage and traction retinal detachment. These features resemble those of human type 2 diabetes with insulin hyposecretion and make SDT rats an acknowledged model for studying type 2 DR.

Due to the lack of models mimicking the proliferative stage of DR, researchers have turned to non-diabetic animal models, and, in particular, the oxygen induced retinopathy (OIR) model that very closely recapitulates the pathologic events occurring in ROP [24]. In this model, one-week-old mouse pups are exposed to hyperoxia, which obliterates capillaries in the retina. Upon return to room air, the retina becomes hypoxic and triggers a vascular repair response, which then results in the formation of neovascular tufts towards the vitreous, a hallmark of ischemic retinopathies in human pathologies. The tuft formation is often referred to as ‘pathological angiogenesis’ and has made the OIR model a key tool in addressing vascular pathology in ischemic retinopathies. Among the models mirroring subretinal neovascularization, the mouse model in which CNV is induced by laser treatment is one of the best models currently used to mimic the pathologic mechanisms in nAMD, although some differences in the chorio-retinal environment and in the disease state (acute versus chronic) have been evidenced between mice and humans. In this model, a thermal insult disrupts Bruch’s membrane leading to an inflammatory/wound-healing response and concomitant CNV in which newly formed choroidal blood vessels grow into the subretinal compartment [25]. As in nAMD, the choroidal capillaries are explicitly involved in the neovascular response, although the more extensive injury to Bruch’s membrane represents a more powerful angiogenic stimulus than likely occurs in nAMD.

Alongside to ischemic retinopathies, degenerative diseases of the retina also include inherited retinal dystrophies. Over the last years, mutant animal models have greatly contributed to our understanding of the molecular mechanisms underlying this class of diseases, although they do not always mimic the retinal phenotype observed in humans. Genetic in vivo models of inherited photoreceptor degeneration are characterized by mutations spanning in numerous genes. Among these models, rd10 mice show a mutation in exon 13 of the beta subunit of the rod cGMP phosphodiesterase gene that results in photoreceptor degeneration [26]. Rd10 mice provide a good model for studying the pathogenesis of RP in humans and are considered to replicate human RP better than rd1 mice (an additional model of RP based on a different mutation of the rod cGMP phosphodiesterase gene), because of its later onset and milder retinal degeneration. Photoreceptor degeneration in rd10 mice is associated with an increased expression of inflammatory genes [27] and an ample piece of literature data converges in highlighting a critical role for inflammation in the pathogenesis and/or in the progression of retinal degenerative pathologies (for Ref., see [28]).

## 4. The uPAR System

Drugs targeting pathways common to different eye diseases may open up a very general and widely applicable approach for therapeutic interventions. Here, we will bring major evidence that the uPAR system is emerging as a key player at the intersection between angiogenesis, inflammation and neurodegeneration thus arising as a good candidate target to counteract degenerative diseases of the retina.

Stroma invasion by proliferating endothelial cells involves the activation of proteolytic enzymes required to degrade the endothelial basement membrane and extracellular matrix (ECM), thus allowing endothelial cell migration through the lysed matrix proteins [29]. Among the protease systems involved in angiogenesis, a central role is played by a system formed by urokinase-type plasminogen activator (uPA) and its receptor uPAR. uPAR is produced as a 313 amino acid protein devoid of the transmembrane and cytoplasmic domain. uPAR is indeed anchored to the plasma membrane by a glycosyl phosphatidylinositol moiety; during the glycolipid modification of uPAR, a C-terminal sequence is removed, generating a fully processed uPAR that contains residues 1–283 [30]. Cell-surface uPAR is formed by three domains (D1, D2 and D3) that form a globular structure delimiting a central pocket in which the ligand binding domain is located [31]. uPAR participates in the regulation of the peri-cellular proteolysis thus activating a cascade of proteolytic events that leads to the degradation of ECM [32]. In addition, the uPAR surface that is not involved in the binding to ligands is available to interact with integral membrane proteins acting as co-receptors; in many cases, the interaction between uPAR and its co-receptors is mediated by the uPAR88–92 sequence located at the linking region between the domains D2 and D3 [33,34]. Through the lateral interaction with transmembrane proteins possessing intracellular domains, uPAR is capable of influencing intracellular signal transduction and to participate thereby in regulatory mechanisms within the cell. Through these interactions uPAR can be part of dynamic multi protein signaling complexes, which are in the literature conventionally designated as the “uPAR interactome” [35]. There is a broadly-based ongoing research assessing the composition and biological function of these complexes in different physiological and pathological processes. In this respect, the uPAR system plays a broader role in multiple stages characterizing several pathological conditions and, in particular, is a key factor for the invasive capacity of malignant tumors. Table 1 summarizes some of the main diseases associated to upregulated uPAR, which represents an effective biomarker of disease progression in organs other than the eyes. Later sections of this review will recapitulate recent findings on the uPAR system and its functional role in eye pathologies.

Beside the membrane anchored uPAR, the soluble form of the receptor ((s)UPAR) possesses regulatory functions. (s)UPAR is generated by the proteolytic cleavage of the membrane anchored uPAR and retains most of the uPAR activities; similarly to uPAR, (s)uPAR is involved in cell attachment, motility and migration (for Ref., see [48]) and elevated plasma (s)uPAR is considered as a biomarker in several chronic inflammatory diseases including cancer, cardiovascular diseases, chronic kidney diseases and diabetes [49]. In respect to eye diseases, a recent study demonstrates that plasma levels of (s)uPAR are significantly increased in patients suffering from nAMD suggesting that chronic inflammation may be involved in its pathogenesis [50]. An additional study associates high plasma (s)uPAR levels with the progression of Behçet’s disease, a chronic, systemic vasculitis affecting many systems, characterized by ocular inflammation indicating that (s)uPAR may be considered a good marker of inflammatory diseases of the eye [51].

Among uPAR ligands also including structurally unrelated proteins, uPA is the major endogenous ligand. uPA is produced and secreted by many cell types, including endothelial cells. It is a serine protease involved in the conversion of inactive plasminogen into active plasmin. After secretion as a single polypeptide chain precursor of 411 amino acids with the C-terminus containing a catalytic serine protease domain, pro-uPA is converted into the active two-chain form by plasmin in a positive feedback loop. After the two-chain uPA is cleaved by a second round of proteolysis, a single chain form of uPA, which is about 250 times more active than the two-chain form, is generated together with an inhibitory amino-terminal fragment (ATF) (for Ref., see [52]). ATF binding to uPAR affects the interaction between uPA and uPAR with a consequent inhibition of the functional effects of uPAR activation (for Ref., see [53]). uPA binding to uPAR increases the activation of plasminogen into plasmin that is involved in the dissolution of ECM and basement membrane during tissue degradation (for Ref., see [52]). In particular, plasmin generated by uPA can breakdown ECM either directly or indirectly by activating matrix metalloproteinases that act as proteolytic cleavers of the ECM components. In turn, degradation of ECM results in the release of ECM-bound growth factors, which act as a positive feedback loop thus enhancing the expression of different components in the uPAR system [54]. Participation of uPA to eye pathologies has been less investigated except for some results demonstrating that uPA levels modulate RGC degeneration through the regulation of the intraocular pressure (IOP) suggesting that regulating uPA may be regarded as a potential strategy to attenuate RGC death in response to elevated IOP [55,56].

## 5. uPAR-Co-Receptor Interaction

Despite lacking any transmembrane or intracellular domain, uPAR additionally acts as initiator of a diversity of intracellular signal transduction cascades including chemotaxis, invasion, proliferation, and survival. Intracellular uPAR signaling can be traced back to multiple interaction partners among which the first identified has been the formyl peptide receptor (FPR) 1, followed by FPR2 and FPR3 [57,58]. FPRs are members of the family of G protein-coupled receptors and there is accumulating evidence that they are major players in both angiogenic and inflammatory processes [59,60] with recent data indicating an important role for FPRs in an increasing range of human diseases in which inflammatory processes are recognized as critical components [61]. FPRs have been shown to participate to the pathogenesis of several eye pathologies in which they play a major pro-inflammatory role. In animal models of DR, for instance, high glucose has been shown to increase FPR2 levels thus exacerbating Müller glial cell chemotaxis, proliferation and VEGF production, therefore, contributing to the progression of PDR [62]. A major role of FPRs in pathological angiogenesis has been suggested by recent results demonstrating that FPR inhibition reduces the neovascular and inflammatory response elicited by the vitreous of patients with PDR in in vitro and in vivo assays thus indicating that FPR activation may play a role in neovessel formation during PDR (for Ref., see [63]).

A second class of uPAR lateral partners is represented by integrins that seem to possess important regulatory effects in multiple pathological conditions with angiogenic and inflammatory components [64]. Integrins are transmembrane cell surface glycoprotein heterodimers formed by α- and β-subunits linking ECM to the cytoskeleton thus conferring specificity to uPAR signaling, with different integrin sub-types activating distinct intracellular signaling pathways [65]. However, defining how uPAR and integrins work together is a matter of debate with still unresolved questions of whether uPAR interacts directly with integrins or activates integrin signaling by enhancing cell-ECM contacts through uPAR binding to vitronectin, an ECM component acting as an additional ligand for uPAR (for Ref., see [66]). Among integrins, αvβ3 is the primary integrin heterodimer mostly associated with various pathological processes, such as vascular leakage, neovascularization and inflammation [64]. In fact, αvβ3 integrin is upregulated on proliferating endothelial cells during angiogenesis and vascular remodeling [67] and contributes to inflammation by regulating the nuclear factor kappa-light-chain-enhancer of B cells (NF-kB) -induced pro-inflammatory responses [68]. In particular, upregulated levels of αvβ3 integrin are associated to main inflammatory processes in diabetic nephropathy [45], while inflammation-induced expression of αvβ3 integrin regulates astrocyte reactivity [69]. In models of ischemic brain, activation of αvβ3 integrin is associated with the release of inflammatory factors whereas its inhibition has been shown to reduce inflammatory processes [70,71]. Both experimental and clinical evidence demonstrate that integrins play a role in the pathogenic processes of DR and nAMD [72]. In particular, pharmacological inhibition of the integrins αvβ3, αvβ5 and α5β1, which are the main integrins implicated in DR- and nAMD-associated disease processes, has been shown to attenuate VEGF-induced vascular leakage in the mouse retina as well as angiogenesis-induced retinal leakage in the cynomolgus CNV model [73]. Upon activation, αvβ3 integrin initiates a signaling cascade in which an important role is played by rat sarcoma (Ras)-related C3 botulinum toxin substrate 1 (Rac1) that belongs to the Rho proteins, which are a dynamic group of GTPases participating in a multitude of cellular activities and functions. From their effects on the expression of angiogenic factors to their role in the integrin signaling pathways and endothelial cell morphogenesis, Rho proteins prove to be an important part of the angiogenesis machinery [74]. Little is known about uPAR interaction with αvβ3 integrin although there are some indications that αvβ3 integrin is implicated in regulatory functions mediated by the uPAR system. In this respect, uPAR activation by proteoglycan, a major component of ECM, promotes the acquisition of an angiogenic profile by human umbilical vein endothelial cells (HUVEC) through the involvement of αvβ3 integrin [75]. In addition, in VEGF-stimulated HUVEC, inhibiting uPAR blunts αvβ3 integrin activity thus preventing the acquisition of an angiogenic phenotype [33].

Additional lateral partners of uPAR include tyrosine kinase growth factor receptors, as for instance VEGFR2, which interacting with uPAR may participate to VEGF signaling. In particular, among protease-dependent and -independent mechanisms through which uPAR mediates angiogenesis, uPAR coupling to VEGFR2 activates a non-proteolytic signaling pathway that stimulates HUVEC proliferation in response to uPA [76]. VEGFR2 involvement in uPAR signaling has been indirectly demonstrated by recent findings showing that in HUVEC, knockdown of uPAR impairs VEGFR2 signaling and reduces cell proliferation in response to VEGF, while uPAR deficiency in mice prevents retinal angiogenesis in response to VEGF indicating that uPAR-VEGFR2 interaction is crucial for VEGF signaling in endothelial cells [77]. The additional finding that uPAR binding to VEGFR2 leads to uPAR redistribution to the leading edge of migrating endothelial cells thus providing them with the localized proteolytic capacity to invade the surrounding tissue, is indicative of the possibility that uPAR plays an important role in inducing cell migration downstream the VEGF-VEGFR2 axis (for Ref., see [78]).

## 6. Mechanisms of uPAR Signaling

**Figure 2:**
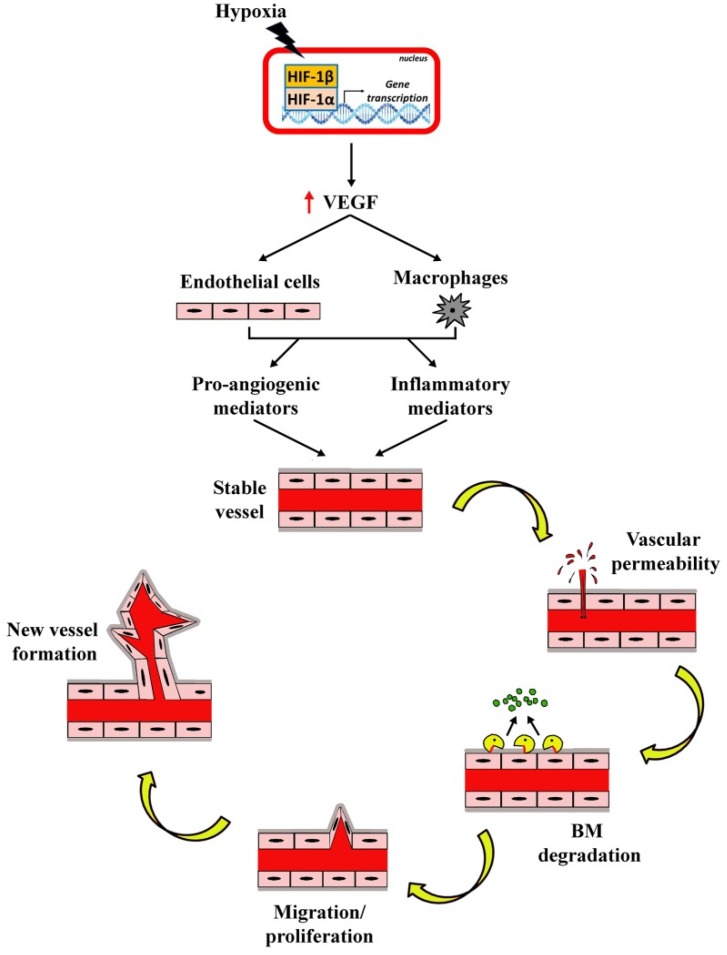
Simplified diagram showing the cascade of events leading to angiogenesis in response to hypoxia. Hypoxia induces the stabilization of the hypoxia inducible factor 1α (HIF-1α) and its consequent binding with HIF-1β. The complex HIF-1α/HIF-1β acts as a transcription factor thus resulting in increased expression of pro-angiogenic factors. Among them, the vascular endothelial growth factor (VEGF) acts on several target cells of which retinal and choroidal endothelial cells and macrophages are among those promoting the release of pro-angiogenic and inflammatory mediators [80,81]. Beside these cells, local cells at the retina including pericytes, microglia and Müller cells also respond to increased VEGF levels [81,82,83]. They act on stable vessels to enhance vascular permeability and to promote basement membrane (BM) degradation and endothelial cell migration/proliferation finally leading to new vessel growth.

Hypoxia/ischemia is one of the main players in switching on vascular complications by activating oxygen-sensitive transcription factors that trigger the production of pro-angiogenic/inflammatory mediators among which VEGF is the main regulator of angiogenesis in physiological and pathological conditions and is the major growth factor mediating vascular leakage [79]). A simplified representation depicting the essential steps in the angiogenic cascade is shown in Figure 2. Under the mechanisms underlying the angiogenic process, hypoxia initiates the cascade of cell division through several pro-angiogenic and inflammatory mediators, whereas cell migration is mediated by proteolytic degradation of ECM components.

Among the oxygen sensing transcription factors hypoxia-inducible factor 1 (HIF-1) is a heterodimer composed of two subunits: a labile HIF-1α subunit and a stable HIF-1β subunit. Under normoxia, HIF-1α is continuously degraded by proteasome, but when oxygen becomes limiting, HIF-1α escapes degradation, accumulates in the cell, is imported into the nucleus, dimerizes with HIF-1β and activates dozen of genes encoding for a multitude of pro-angiogenic and inflammatory factors that are involved in both excessive vasopermeability and neovessel growth [84]. HIF-1 also activates the expression of genes related to ECM degradation (for Ref., see [85]) and modulates the expression of genes involved in autophagy, apoptosis, redox homeostasis and immunity [86], thus suggesting a role as a multiplicative factor in mediating an abundance of pathological responses to an ischemic insult.

When cells are exposed to pro-angiogenic or inflammatory factors, the signal transducer and activator of transcription 3 (STAT3) becomes phosphorylated, homodimerizes and then moves from the cytoplasm to the nucleus where it triggers the transcription of genes involved in angiogenesis and inflammation including HIF-1 itself [87]. Additionally, NF-kB and cAMP-responsive element binding protein (CREB) are both activated by inflammation and, as a consequence, NF-kB dimerizes while CREB is phosphorylated and both translocate into the nucleus where they recruit transcriptional co-activators to induce the expression of a large array of cytokines thus reinforcing inflammatory processes (for Ref., see [88,89]).

Among pro-angiogenic factors, VEGF exerts its effects by stimulating endothelial cell proliferation, migration and vessel formation, acting through its paralog receptors (VEGFRs) [90]. In addition, VEGF overexpression is correlated with BRB breakdown in animal models of neovascular retinal diseases and in patients suffering from DR or nAMD [22]. In DR, for instance, VEGF accumulates very early and its inhibition at later stages is mostly intended to regulate excessive vasopermeability and consequent DME [91]. In addition, VEGF has been identified as a major factor leading to nAMD and anti-VEGF therapies have become a major strategy to counteract this pathology [92]. Beside VEGF, multiple pro-angiogenic factors are involved in endothelial cell proliferation directly or in synergy with VEGF itself, thus playing an important role in new vessel growth and vasopermeability excess [93]. In DR, for instance, angiopoietin-2 plays an important role in the regulation of high glucose-associated alterations of vascular permeability, presumably through a combined action with VEGF [94].

In addition to pro-angiogenic factors, inflammatory cytokines mediate a broad range of biological processes leading to new vessel growth and BRB dysfunction [95]. Upon cytokine stimulation, endothelial cells secrete adhesion molecules resulting in leukocyte adhesion to the retinal capillaries, a process that impairs the vascular wall integrity thus contributing to increased vascular permeability [96]. Cytokine production stimulates the release of additional inflammatory mediators by Müller cells, thus creating a positive feedback loop that contributes to BRB breakdown and vessel leakage [13]. Increasing evidence indicates that the accumulation of inflammatory cytokines in the vitreous may contribute to DR progression and may explain why about 30% of DR patients fail to respond to anti-VEGF treatments [97]. The additional finding that inflammatory processes are an early marker in vascular diseases of the eye may explain why anti-inflammatory drugs may have beneficial effects as preventive or adjunctive therapies in patients who do not respond to conventional anti-VEGF therapy [98].

As discussed so far ischemic retinopathies are characterized by increased vasopermeability and/or neovessel growth in response to hypoxia, while an additional devastating disease of the retina, RP, is instead characterized by low oxygen consumption generating a hyperoxic retinal environment, when rods, which represent about 95% of photoreceptors, degenerate [99]. As a consequence, elevated oxygen tension on the one hand would induce the regression of preexisting blood vessels and, on the other hand, it would also exert toxic effects ultimately leading to cone death [100]. In addition, cone degeneration involves major inflammatory events as demonstrated by elevated levels of inflammatory cytokines identified in the vitreous humor from RP patients [101]. In addition, in RP models, microglial activation with a consequent increased release of inflammatory cytokines has been demonstrated [28,102] while suppression of the gliotic response of Müller cells is effective in slowing down photoreceptor degeneration [103]. Finally, inhibiting NF-kB and STAT3 reduces the production of inflammatory cytokines and prevents photoreceptor degeneration suggesting that these transcription factors act as master regulators of the degenerative processes affecting cones [104].

**Figure 3:**
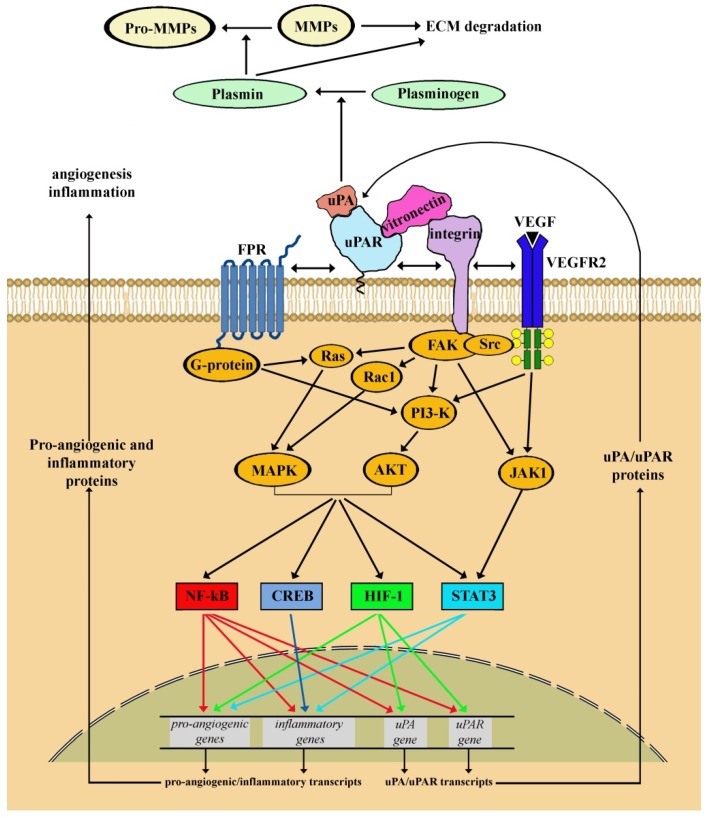
Schematic representation of signaling pathways downstream urokinase-type plasminogen activator (uPA) receptor (uPAR) and its co-receptors. uPAR is anchored to the plasma membrane and binds specifically to uPA. uPA can catalyze the process from plasminogen to plasmin. Plasmin cleaves and activates matrix metalloproteinases (MMPs). Both plasmin and MMPs degrade many extracellular matrix (ECM) components. Extracellularly, the binding of uPAR with vitronectin promotes cell adhesion and migration. Intracellularly, due to the lack of transmembrane and cytosolic domains, uPAR binds to lateral partners for signal transduction thus resulting in the activation of multiple signaling pathways that mediate a variety of cellular responses. In particular, uPAR binding to formyl peptide receptors (FPRs), a family of G protein-coupled receptors, activates the phosphatidylinositol 3-kinase (PI3K)/protein kinase B (AKT) pathway thus promoting the proliferative process. Additional, uPAR/integrin interaction promotes the activation of focal adhesion kinase (FAK), a cytoplasmic tyrosine kinase that plays a critical role in integrin-mediated signal transduction. Once activated, FAK turns on signaling pathways including the rat sarcoma (Ras)/mitogen activated protein kinase (MAPK), Ras-related C3 botulinum toxin substrate 1 (Rac1)/MAPK, PI3K/AKT and janus kinase 1 (JAK1) pathways, which are involved in directing cellular responses to a wide array of stimuli. In addition, FAK activation by uPAR/integrin recruits proto-oncogene tyrosine-protein kinase (Src) family tyrosine kinase to enhance the phosphorylation of VEGF receptor 2 (VEGFR2) in response to VEGF, which in turn activates both the PI3K/AKT and the JAK1 pathways. Signaling pathways downstream to uPAR interaction with its lateral partners concur to activate transcription factors including nuclear factor kappa-light-chain-enhancer of activated B cells (NF-kB), cAMP response element-binding protein (CREB), HIF-1 and signal transducer and activator of transcription 3 (STAT3) of which NF-κB, HIF-1 and STAT3 activate the transcription of pro-angiogenic and inflammatory genes, while CREB is involved in the transcription of inflammatory genes. In addition, both NF-κB and HIF-1 mediate the transcription of the uPA/uPAR genes.

There are several targets that trigger the signaling pathways in response to an ischemic insult or a gene mutation in ocular pathologies. Among them, we will discuss here the involvement of the uPAR system in the molecular cascade leading ultimately to the activation of transcription factors that regulate the expression of genes coupled to angiogenic/inflammatory responses [105]. A summary representation of signaling pathways involving uPAR and its co-receptors is shown in Figure 3.

Much information about uPAR signaling in angiogenic processes derives from studies in tumor angiogenesis in which the synergism among different kinases downstream uPAR results in a high degree of signal amplification to activate angiogenic/inflammatory processes [105]. Among them, the activation of the focal adhesion kinase (FAK), a cytoplasmic tyrosine kinase that plays a critical role in integrin-mediated signal transduction, represents a priming step in inducing kinase-mediated cascades including the Janus kinase 1 (JAK1), phosphatidylinositol 3-kinase (PI3K)/protein kinase B (AKT), Ras/mitogen activated protein kinase (MAPK) and Rac1/MAPK pathways, which are involved in directing cellular responses to a wide array of stimuli by increasing the expression of sets of pro-angiogenic and inflammatory genes [106]. For instance, the cooperation of uPAR with the integrin/FAK pathway promotes endothelial cell proliferation and migration by specific proteoglycans, which have important effects on various aspects of angiogenesis [75]. Additionally, FAK recruits members of the proto-oncogene tyrosine-protein kinase Src family to enhance the activity of VEGFR2 that, in turn, induces angiogenic profiles by activating the PI3K/AKT, MAPK and JAK1 pathways [107,108]. Signaling pathways downstream uPAR interaction with its lateral partners concur to activate different transcription factors including NF-κB, CREB, HIF-1 and STAT3 of which NF-κB, HIF-1 and STAT3 activate the expression of pro-angiogenic and inflammatory genes, while CREB is involved in the transcription of inflammatory genes [109,110,111]. In particular, the JAK1 pathway mediates the phosphorylation of STAT3 likely through VEGFR2 activation [107] while the PI3K/AKT pathway participates to uPAR coupling to the inflammatory cascade through the activation of NF-κB (for Ref., see [112]). In addition, overexpressed uPAR leads to an increased transcription of the uPA gene through MAPK activation consequent to uPAR interaction with FPRs and integrins thus enhancing the cascade of proteolytic events that leads to the active degradation of ECM components [32]. Finally, both NF-kB and HIF-1 bind to cognate sequence elements of uPA and uPAR promoters thus mediating the transcription of uPA and uPAR genes and participating to the upregulation of the uPAR system in response to hypoxia [113,114,115,116]. Most interestingly, uPAR may undergo translocation into the nucleus where acquires transcriptional activity through its association with different transcription factors to regulate the expression of multiple genes including its own gene thus indicating that uPAR may function as a direct activator of gene transcription able to promote the initiation of a positive feedback loop that drives the cellular response to an insult [117]. Little is known about the signaling pathways underlying uPAR-mediated angiogenic processes in neovascular eye diseases with the exception of some findings in VEGF-stimulated retinal endothelial cells in which uPAR has been found to mediate the activation of HIF-1 and STAT3 through the involvement of several kinases including JAK1 and members of the MAPK family [108].

## 7. The uPAR System in the Diseased Eye

In the eye, the uPAR system has been found to participate in many pathologies and its dysregulation plays a major role in ocular diseases. However, the spectrum of action of the uPAR system has been recently extended far beyond its classical proangiogenic function and has emerged as a central actor in inflammatory processes of the eye.

### 7.1. Ischemic Retinopathies

In general, uPAR is expressed at low levels in healthy conditions and becomes upregulated in pathological states of the eye. In response to hypoxia, uPAR is overexpressed by retinal endothelial cells in which it is mostly localized to proliferating capillaries extending into the vitreous cavity thus playing an important role in the acquisition of angiogenic phenotype [118]. In the choroid, uPAR is localized to endothelial cells where it is upregulated in response to laser treatment indicating a close association with CNV [119,120]. The additional finding that in rodent models of neovascular eye diseases, blockade of overexpressed uPAR results in reduced vessel proliferation in concomitance with decreased VEGF accumulation suggests that uPAR upregulation may stimulate angiogenesis by enhancing VEGF production [119,121]. Interestingly, VEGF has been shown to activate the conversion to uPA by its precursor pro-uPA thus creating a feedback loop that positively participates to the angiogenic process [122]. uPAR upregulation has been also demonstrated in rodent models of DR in which uPAR participates to the generation of a proteolytic cascade that has detrimental effects on BRB integrity [123,124,125,126]. In a comprehensive multiple analysis to identify angiogenic factors associated with PDR, overexpressed uPAR has been determined in the vitreous humor of DR patients suggesting that it may be involved in PDR pathogenesis [127].

Among the lateral partners of uPAR, FPRs are expressed by RPE cells, iris cells and retinal endothelial cells [128,129,130] although some findings have evidenced FPR localization to the neuroretina [131]. Increased FPR expression has been determined in the iris of a mouse model of RI [128], in the retina of rat models of DR [124] and in the choroidal tissue of a mouse model of CNV [119]. A population-based study performed in a Chinese cohort demonstrated a genetic association of an inflammation-related gene, FPR1, with exudative AMD [132]. In addition, upregulated FPRs have been determined in the retina of PDR patients although the clinical significance of FPR overexpression remains to be determined [62]. Interestingly, using angiogenesis models, Rezzola et al. have demonstrated that antagonizing FPRs counteracts the pro-angiogenic/inflammatory activity exerted by the vitreous fluid from PDR patients, thus suggesting that FPRs may play a role in the pathogenesis of neovascular eye diseases, although FPR function has not been fully elucidated yet [133].

Of the uPAR co-receptors, integrins are not generally expressed on quiescent microvessels, but are selectively upregulated by proliferating blood vessels in response to pro-angiogenic growth factors indicating that they may play a crucial role in vascular eye pathologies. In both OIR and CNV models, blocking integrins inhibits neovessel growth presumably through inhibiting MMP expression and promoting the apoptosis of proliferating vascular cells, both events concurring to reduced neovascularization [134]. In addition, in DR models, integrins have been shown to participate to high glucose-induced alterations of ECM signaling thus compromising the integrity of the basement membrane structure and contributing to vascular permeability and vessel proliferation [135]. Among integrins, findings about the involvement of αvβ3 integrin in ocular neovascular diseases can be traced back to 1996 when Friedlander et al. extended their earlier studies about αvβ3 integrin role in tumor angiogenesis [136] to the demonstration that αvβ3 integrin is indeed upregulated in neovascular ocular tissues from patients with PDR or nAMD [137]. In addition, in the OIR model, αvβ3 integrin upregulation has been demonstrated in neovascular endothelial cells supporting the possibility that increased expression of αvβ3 integrin likely contributes to retinal neovascularization [138]. In a model of iris neovascularization, αvβ3 integrin is upregulated in endothelial cells of both pre-existing and newly formed iris vessels [139]. Evidence that αvβ3 integrin is important in ocular angiogenesis is also provided by studies demonstrating that αvβ3 integrin antagonism is effective in reducing pathologic angiogenesis possibly through inhibiting the VEGF/VEGFR2 axis [140,141]. In particular, intravitreal administration of a mimetic peptide derived from collagen IV appears to ameliorate ocular neovascularization and vascular leakage through reduced binding of αvβ3 integrin to VEGFR2 [142]. Interestingly, among a panel of integrins known to regulate endothelial cell function only αvβ3 integrin is expressed in the endothelium from retinal specimens of PDR patients thus suggesting a key role in PDR pathogenesis [143].

An additional lateral partner of uPAR is VEGFR2 that is the major driver of VEGF-induced angiogenic and inflammatory processes although its role in uPAR signaling is less known. In this regard, the ligand uPA has been demonstrated to mediate the pro-angiogenic effects of VEGF by inducing VEGF receptor expression (for Ref., see [144]). In eye diseases, there is only limited evidence about VEGFR2 involvement in the pathogenetic role of uPAR with indirect demonstrations that the interaction of uPAR with VEGFR2 may promote VEGF-induced angiogenesis in in vitro and in vivo models of neovessel formation [77]. Additional findings using uPAR inhibitors demonstrate that reduced activation of VEGFR2 leads to decreased neovascular response in both the OIR model of neovessel growth and retinal endothelial cells proliferating in response to VEGF thus suggesting the possibility that uPAR may interact with VEGFR2 to activate pathologic processes in the diseased eye [108,121].

### 7.2. Retinitis Pigmentosa

In contrast to the increased expression of the uPAR system that is generally coupled to inflammation in the ischemic eye, RP is instead characterized by drastically low levels of both uPAR and uPA as demonstrated recently in the rd10 model [145]. The additional finding that stabilizing HIF-1α results in recovering retinal levels of uPA and uPAR indicates that the expression of uPA/uPAR in the retina is correlated with the activity of HIF-1 in line with previous studies in models of tumor angiogenesis [114,115]. Of the uPAR co-receptors involved in RP, FPRs display stable levels although in the presence of a negligible amount of uPA/uPAR. In contrast, the activity of the αvβ3 integrin/Rac1 pathway is drastically increased thus indicating integrin involvement in the inflammatory cascade triggered by photoreceptor degeneration. This is in line with previous findings demonstrating that in a mouse model of RP, blocking αvβ3 integrin improves morphological and functional parameters of photoreceptor degeneration through inhibition of microglial phagocytosis [146]. In addition, reducing Rac1 activity has been shown to increase the survival of photoreceptor cells and to rescue rod function by modulating oxidative stress [147].

## 8. Inhibition of the uPAR System

Animal models have been widely used to obtain the most important knowledge advances in many biological fields and have given scientific support to new therapeutic approaches for human diseases. In fact, the possibility to interfere with a specific pathway using pharmacologic or molecular approaches makes the animal models a powerful tool in unraveling the mechanisms of human physiopathology.

### 8.1. Pharmacological Approaches to Inhibit the uPAR System

Earlier results about the possibility to reduce neovessel formation in the retina by pharmacological interaction with the uPA/uPAR system can be traced back to 2003 when Le Gat et al. have demonstrated anti-angiogenic efficacy of intravitreal delivery of ATF in a mouse model of OIR. As ATF binds to uPAR on the cell surface, it blocks the interaction between uPA and uPAR thus inhibiting uPA/uPAR-dependent neovascular tuft formation [53]. This study was the proof of concept that molecules disrupting the interaction between uPA and uPAR might be effective in counteracting retinal neovascular pathologies. Results from additional studies confirmed that reducing the activity of the uPAR system using peptide inhibitors may be a strategy for counteracting pathological angiogenesis and microvascular leakage. For instance, administration of Å6, an 8-amino acid peptide, inhibits retinal neovascularization in a mouse model of OIR [118]. Å6 is also able to inhibit CNV in mouse or monkey models of nAMD [120,148], whereas, in rat models of DR, it prevents the increase in microvascular permeability by inhibiting the proteolytic degradation of the vascular endothelial-cadherin, a junctional protein that is generally associated to increased vascular permeability in response to pro-angiogenic stimuli [126]. In addition, Å6 inhibits the migratory and invasive capacity of retinal endothelial cells in response to hepatocyte growth factor, a cytokine known for its pro-angiogenic properties [149].

Since 2005, the observation that the residue Ser90 plays a critical role in uPAR signaling and that its substitution with a Glu residue results in inhibiting uPAR activity, has led to the synthesis of a peptide family that blocks the uPAR pathway by interfering with the complex cross-talk involving uPAR, FPRs and integrins [150,151]. Among blockers of the uPAR system, the peptide Ac-L-Arg-Aib-L-Arg-L-α(Me)Phe-NH2, named UPARANT (and recently designated as Cenupatide by the WHO as the International Nonproprietary Name) prevents formyl peptide binding to FPRs, an indication that formyl peptides and UPARANT share the same binding site. In angiogenesis assays, UPARANT blocks VEGF-triggered signaling to reduce endothelial cell proliferation, motility and tube formation by binding with high affinity to FPRs and with lower affinity to integrins [34,108]. In addition, UPARANT may also directly bind to αvβ3 integrin thus preventing integrin receptor activation without binding to uPAR or interfering with the uPA/uPAR binding [33,34].

**Figure 4:**
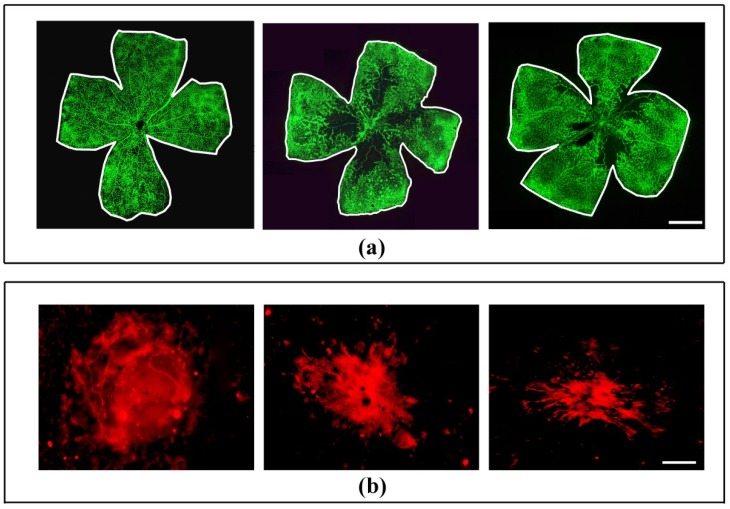

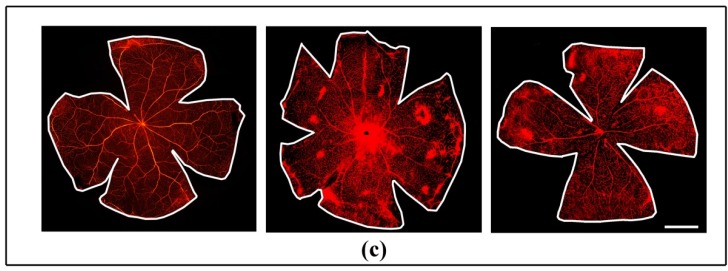
The uPAR-derived peptide UPARANT mitigates angiogenesis and blood retinal barrier (BRB) breakdown in rodent models of ocular pathologies. Panel (**a**) shows representative images of flat-mounted retinas immunolabeled with a rat monoclonal antibody directed to CD31 from the OIR model. Retinas were collected at PD17 in control mice and in OIR mice either untreated or UPARANT-treated. In comparison to a normally developed vasculature, retinal vasculature in response to hypoxia is characterized by a large avascular area in the central retina and numerous engorged neovascular tufts. Intravitreal UPARANT at 1.5 mg/mL reduced drastically vessel tuft formation, but did not influence the extent of the avascular area. Images in Panel (**a**) are from unpublished material. They were collected from retinas that were processed for CD31 immunohistochemistry as detailed in Figure 1. Scale bar: 1 mm. Panel (**b**) shows choroidal neovascularization (CNV) in the laser-induced CNV model. CNV was detected using an antibody directed to CD31 in flat-mounts of RPE-choroid complexes from mice intravitreally treated with vehicle, with UPARANT at 4 mg/mL or 12 mg/mL. Scale bar: 100 µm. Images in Panel (**b**) originate from previously published work [119]. Panel (**c**) shows retinal vascular permeability as determined by Evans blue perfusion in control rats and in the streptozotocin (STZ) model of diabetic retinopathy. STZ rats were either untreated or treated systemically with UPARANT at 8 mg/kg. BRB breakdown with leakage of the dye was evident in STZ rats. Treatment with UPARANT reduced dye leakage. Images in Panel (**c**) are from unpublished material. Leakage was evaluated after rat perfusion with 0.5% Evans blue dye into the left ventricle. Scale bar: 1.5 mm.

In in vivo models of angiogenesis, UPARANT has been shown to abrogate neovessel formation induced not only by VEGF, but also by the vitreous fluid from patients with PDR thus blocking the angiogenic potential of a complex mixture of pro-angiogenic and inflammatory factors [121,133]. Since late 2014, the effects of UPARANT have been first investigated in the mouse model of OIR and the mouse model of nAMD and, in later times, in the STZ-treated rat model of DR. In Figure 4, representative selection of images illustrating UPARANT efficacy in models of ocular pathologies is depicted.

**Figure 5:**
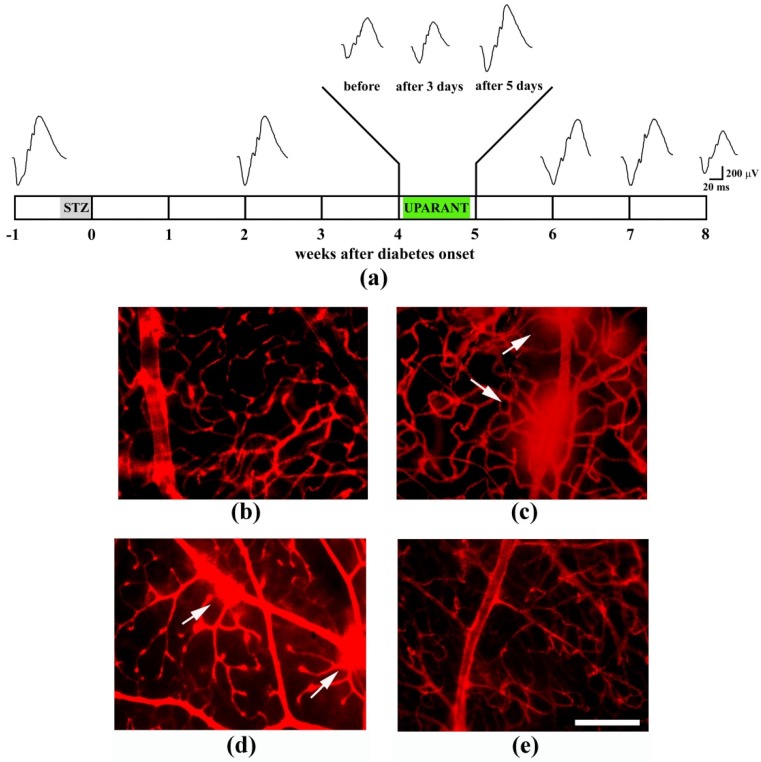
UPARANT recovers dysfunctional electroretinogram (ERG) and blood-retinal barrier (BRB) leakage in the streptozotocin model of diabetic retinopathy. (**a**) Schematic representation of longitudinal ERG monitoring before or at different times after diabetes onset. The arrow indicates the day of diabetes onset. At the fourth week after diabetes onset, ERG becomes dysfunctional. ERG monitoring performed after UPARANT administration demonstrates that ERG amplitude recovers to normal value after 5 days treatment with daily subcutaneous administration of UPARANT at 8 mg/kg (indicated in green). (**b**–**e**) BRB leakage as qualitatively evaluated by Evans blue dye extravasation in control (**b**) and diabetic rats untreated (**c**), systemically treated with either vehicle (**d**) or UPARANT (**e**). UPARANT reduces BRB breakdown. Arrows point to BRB leakage. Scale bar: 200 μm. Adapted from [123].

In the OIR model, intravitreal UPARANT prevents hypoxia-induced retinal neovascularization, inner BRB leakage and visual dysfunction likely by modulating the VEGF/VEGFR2 axis through an inhibitory action at transcription factors regulating VEGF gene transcription, VEGF levels and VEGFR2 phosphorylation [121]. Anti-angiogenic effects of UPARANT are paralleled by an anti-inflammatory action including reduced Müller cell gliosis. In the mouse model of laser-induced nAMD, UPARANT reduces the CNV area and the leakage from the choroid likely by inhibiting transcription factors coupled to pro-angiogenic and inflammatory processes [119]. More recently, in the RI model in which angiogenesis is driven by a VEGF-independent mechanism, intravitreal UPARANT has been found to reduce iris neovascularization by inhibiting upregulated levels of uPA, uPAR and FPR therefore limiting the activation of downstream transcription factors coupled to pro-angiogenic and pro-inflammatory cascades [128]. Further evidence have been added on the efficacy of systemic administration of UPARANT when compared with its intravitral injection in the CNV model in which the ameliorative effects of the drug are almost comparable indicating that UPARANT is taken up by the tissue from the administration site and is conveyed to the posterior segment of the eye by the blood flow [119]. Successively, the effectiveness of systemic UPARANT in counteracting visual dysfunction and BRB leakage have been determined in DR models [123,124]. In a rat model of in type 1 diabetes, in particular, UPARANT when administered when ERG has already become dysfunctional, has been found to recover retinal function by restoring microvascular permeability through a reduced activation of transcription factors that in turn leads to reduced angiogenic and inflammatory processes thus concurring to ameliorate the dysfunctional BRB [123]. Results obtained in a longitudinal ERG study are depicted in Figure 5 in which the effectiveness of UPARANT in recovering both dysfunctional ERG and BRB breakdown can be observed.

**Figure 6:**
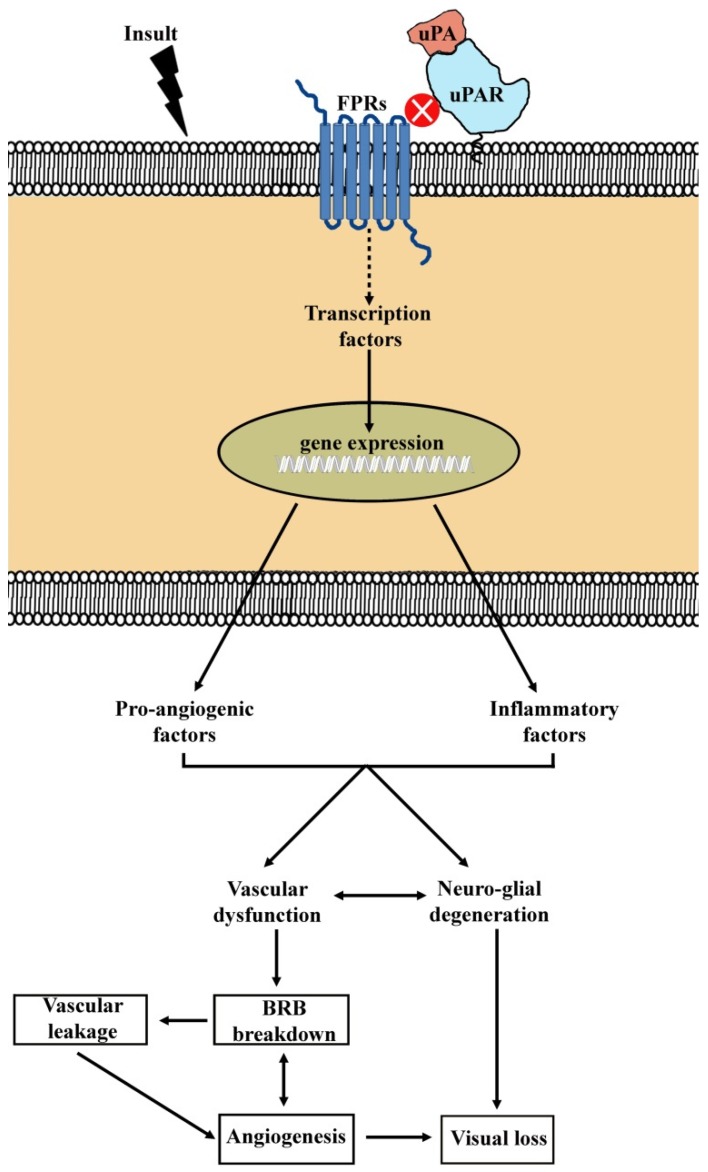
Schematic diagram depicting the hypothetical mechanism by which blockade of the uPAR system results in preserved visual function in the diseased retina. An insult upregulates the protein levels of uPA, uPAR and/or uPAR co-receptors (here represented by FPRs) likely resulting in increased co-receptor signaling that upregulates the expression of pro-angiogenic and inflammatory factors through an increased activation of transcription factors regulating their gene expression. Subsequently, upregulated soluble factors promote vascular dysfunction, thus leading to angiogenesis and/or BRB breakdown, and neuro-glial degeneration, events that all culminate in visual dysfunction. Pharmacological or molecular approaches blocking the activation of the uPAR system recovers the cascade leading to visual loss possibly by preventing the interaction between uPAR and its co-receptors although the exact mechanism of UPARANT action remains to be elucidated.

Overall, these data show that UPARANT acts in a therapeutic regimen by recovering the pathological signs associated to type 1 DR that, on the other hand, is less widespread as compared to DR associated to type 2 diabetes accounting for more than 80% of DR-associated social impairment. In this respect, additional results obtained in the SDT rat, a model of long-lasting type 2 DR, demonstrate that systemic UPARANT is effective in preventing retinal impairment in response to persisting hyperglycemia not only by switching off the transcription factors coupled to angiogenesis and inflammation, but also by downregulating the levels UPARANT targets thus presumably rendering the treatment more effective than if it would act at the receptor downstream level only [124]. Additional action of UPARANT includes preventive effects on Müller cell gliosis and retinal cell death indicating that the compound may contribute to maintaining retinal integrity and providing evidence that long term inhibition of the uPAR system may be a strategy to protect the retina from further worsening of the pathology and slowing down DR progression. Figure 6 illustrates schematically the ability of uPAR system blockade to preserve significantly visual dysfunction by substantially counteracting the molecular cascade leading to BRB leakage and new vessel growth. In fact, UPARANT appears to ameliorate the vascular pathologies of the eye by normalizing the pathological cascade triggered by upregulated levels of uPAR, thus possibly interfering with the interaction of the receptor with its lateral partners.

These findings together concur to demonstrate that among the inhibitors of the uPAR system, UPARANT may be regarded as a significant step forward in the development of new strategies aimed at counteracting eye pathologies characterized by angiogenic/inflammatory profiles as also discussed in a recent paper by Papadopoulos [152].

**Figure 7:**
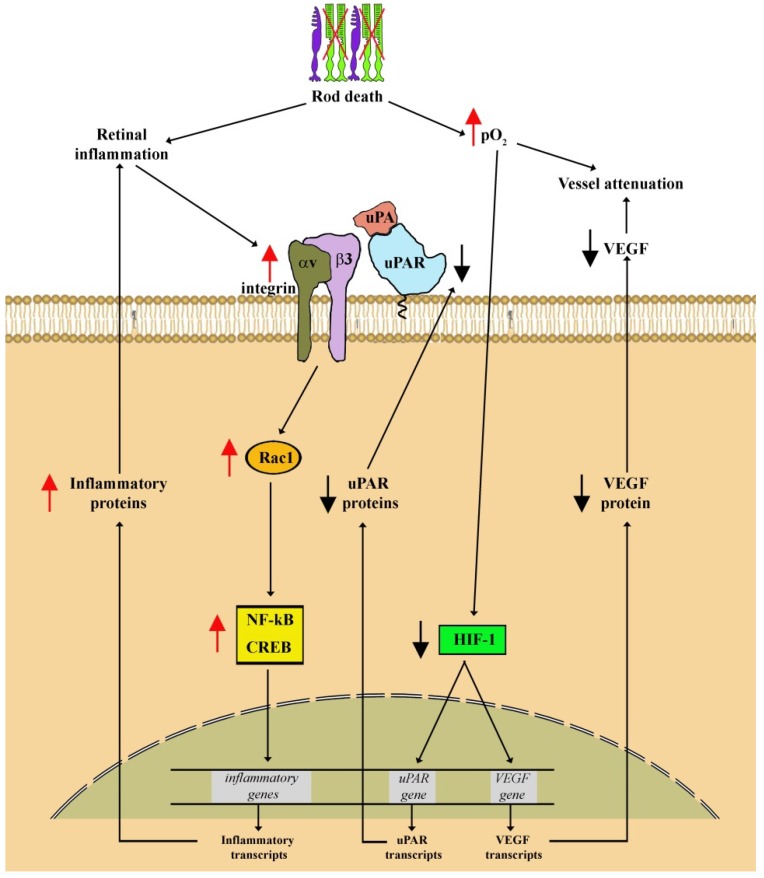
Hypothetical model of uPAR system function in retinitis pigmentosa (RP). The increased retinal levels of oxygen consequent to rod death generates a hyperoxic environment leading to reduced HIF-1 activity. In turn, decreased HIF-1 transcriptional activity downregulates uPAR and VEGF transcripts leading to reduced levels of uPAR and VEGF that presumably lead to vessel attenuation, a feature of RP. At the same time, the inflammatory milieu generated by rod degeneration increases αvβ3 integrin that, acting through multiple intracellular signaling including Rac1, regulates the transcription of different genes including those encoding inflammatory factors that are, in turn, coupled to αvβ3 integrin expression thus exacerbating the inflammation process.

Recent results in the rd10 mouse model of RP deserve special mention. Cammalleri et al. have demonstrated that UPARANT ameliorates cone degeneration and visual dysfunction although in the presence of negligible amount of uPAR [145]. In the RP model, retinal rescue is likely to result from major inhibition of Müller cell activation that presumably breaks the positive feedback loop between Müller cell gliosis and inflammatory drive. The finding of UPARANT efficacy in RP paves the question of whether UPARANT may act directly on co-receptors without binding to uPAR or interfering with uPA/uPAR binding [33,34]. This possibility is supported by the finding that in the absence of FPRs, UPARANT may bind to the cell surface at picomolar concentrations in an integrin-dependent manner [34]. The schematic representation of Figure 7 shows how hypothetically the uPAR system may regulate inflammatory processes in the RP model.

### 8.2. Genetic Approaches to Inhibit the uPAR System

In addition to peptide inhibitors, molecular approaches such as uPA/uPAR gene deletion or the use of antisense oligodeoxyribonucleotides to downregulate uPAR gene expression have been also tested. For instance, results in uPA^−/−^ mice allow to delineate a novel mechanism that contributes to the regulation of endothelial cell proliferation through uPA-dependent de-repression of VEGFR1 and VEGFR2 gene transcription [144]. In additional in vivo models of neovascular eye pathologies, uPAR deletion/silencing has been shown to abrogate neovessel formation in response to hypoxia [118,153] and BRB leakage in response to high glucose indicating an involvement of the uPAR signaling in high glucose-associated retinal damage [125]. On the other hand, there are findings demonstrating that mice with deletion of the uPAR gene exhibit robust angiogenic response in experimental nAMD suggesting that uPAR is not a decisive factor in CNV [154]. The authors of this study rather hypothesize a more important role for the involvement of plasminogen- and/or plasmin-mediated proteolysis. This is underpinned by the findings that no significant CNV develops in mice deficient for uPA or plasminogen.

### 8.3. Inhibition of uPAR Lateral Partners

Results about the role of lateral partners of uPAR are scarce with the exception of major findings about the role of integrins, and αvβ3 integrin in particular, in preclinical studies using animal models of retinal or choroidal neovascularization. In these models, inhibiting the αvβ3 integrin results in reduced ocular angiogenesis and vascular leakage likely by blocking the formation of supramolecular complexes with uPAR thus reducing their downstream intracellular signaling (for Ref., see [73]). Although there are no integrin inhibitors currently available on the market for ophthalmic applications, some clinical trials using ALG-1001, an inhibitor of αvβ3 integrin, have been recently completed and their results demonstrate that the compound may be effective as monotherapy for treatment of either DME or nAMD patients with an efficacy that is not inferior to bevacizumab (ClinicalTrials.gov Identifier: NCT01749891; ClinicalTrials.gov Identifier: NCT02153476; ClinicalTrials.gov Identifier: NCT02348918). Overall, these results suggest that the inhibition of αvβ3 integrin may provide an alternative option to anti-VEGF drugs in treating advanced forms of DR or nAMD. The fact that ALG-1001 should be repeatedly delivered intravitreally, however, does not prevent one of the main limitations of the anti-VEGF therapies.

## 9. Conclusion and Future Perspective

uPAR participates to the proteolytic cascade involved in the regulation of pericellular proteolysis and is also capable of influencing intracellular signal transduction through indirect (with mediation of its ligand uPA) or direct (via lateral contact of its domains) interaction with transmembrane proteins possessing intracellular domains. Here, we have described the uPAR system as an additional pathway in angiogenesis and microvascular dysfunction of the eye, and we propose that its upregulation is responsible for major vascular pathologies. We have also shown that inhibiting upregulated levels of uPAR is effective in counteracting neovessel formation and excessive vasopermeability by reducing increased production of pro-angiogenic and inflammatory mediators. On the other hand, we have also discussed the possibility that dysregulated uPAR system has profound biological consequences on eye physiology with diverse effects whether the system is upregulated or set down. In fact, we have demonstrated that in the RP model, uPAR contribution to RP-associated inflammatory process is almost negligible while integrins are likely to play a central role in RP pathophysiology by enhancing the inflammatory state of the retina thus actively contributing to the secondary cone death. As a future perspective, intervening specifically on the uPAR system might help to elucidate the exact role of each player and its associated downstream signaling. For instance, mice deficient in specific targets of the uPAR system may provide for an opportunity to assess the role of its individual components in different eye diseases.

## Figures and Tables

**Table 1:** uPAR-related diseases in organs other than the eye.

Disease	Pathological Signs	References
Cancer	Angiogenesis, tumor cell proliferation, motility and metastasis	for Ref., see [36]
Rheumatoid arthritis	Angiogenesis and inflammation	for Ref., see [37]
Systemic sclerosis	Oxidative stress	[38]
Lupus erythematosus	Inflammation	for Ref., see [39]
Psoriasis	Cell proliferation and invasion	[40]
Alzheimer disease	Inflammation, oxidative stress and altered blood brain barrier	[41,42]
Coronary artery disease	Inflammation, atherosclerosis and aortic dilation	[43]
Pulmonary fibrosis	Inflammation and fibrosis	for Ref., see [44]
Kidney disease	Inflammation, altered vascular permeability, impaired glomerular filtration and fibrosis	for Ref., see [45]
Bone destructive disease	Inflammation	[46]
Endometriosis	Angiogenesis, inflammation and cell proliferation	[47]
